# Predictive value of glycemic gap and stress glycemia ratio among critically ill patients with acute kidney injury: a retrospective analysis of the MIMIC-III database

**DOI:** 10.1186/s12882-023-03278-z

**Published:** 2023-08-01

**Authors:** Wenkai Xia, Chenyu Li, Meisi Kuang, Yu Wu, Lingyu Xu, Hong Hu

**Affiliations:** 1 Department of Nephrology, Jiangyin People’s Hospital Affiliated to Nantong University, 3 Yingrui Road, Jiangsu 214400 Jiangyin, China; 2 Nephrologisches Zentrum, Medizinische Klinik und Poliklinik IV, Klinikum der Universität München, Ludwig-Maximilians-University Munich, Munich, Germany; 3 Department of Nephrology, the Affiliated Hospital of Qingdao University, Qingdao, Shandong China

**Keywords:** Stress hyperglycemia, Non-diabetic, Acute kidney injury, ICU outcome

## Abstract

**Background and aims:**

Acute hyperglycemia has been identified as a risk factor for acute kidney injury occurrence and mortality in various diseases. The aim of the current study was to investigate the relationship between stress-induced hyperglycemia and adverse outcomes in critically ill patients with AKI.

**Methods:**

We extracted clinical data from Multiparameter Intelligent Monitoring in Intensive Care III version 1.4. Blood glucose and glycosylated hemoglobin during the first 24 h of ICU admission were used to calculate glycemic gap and stress hyperglycemia ratio (SHR). The outcomes included ICU mortality and need for renal replacement therapy. The association of the glycemic gap and SHR with outcomes were determined via logistic regression model and receiver-operating curves. The subgroup analysis of patients with and without diabetes was performed separately.

**Results:**

Higher glycemic gap and SHR were observed in patients who had increased need of RRT, higher mortality rates and longer ICU stay. Multivariate analysis demonstrated that higher glycemic gap (OR 1.01, 95%CI 1.00-1.02, *P* = 0.015), as well as SHR (OR 1.32; 95%CI 1.07–1.64, *P* = 0.009), were independently associated with ICU mortality after adjusting for potential covariates. In subgroup analysis, the association of glycemic gap and SHR were only significant in the non-diabetic population as for the outcome of ICU mortality (OR 2.25, 95%CI 1.64–3.08, P < 0.001 and OR 1.99; 95%CI 1.46–2.72, P < 0.001, respectively).

**Conclusions:**

The glycemic gap and SHR might serve as a potential prognostic indicator of ICU mortality in critically ill patients with AKI, especially in the non-diabetic population.

**Supplementary Information:**

The online version contains supplementary material available at 10.1186/s12882-023-03278-z.

## Introduction

Stress-induced hyperglycemia is common among hospitalized patients, especially in critically ill patients requiring intensive care [1]. The glycemia alteration is proposed to be caused by stimulation of counter-regulatory hormones, use of glucocorticoids and failure of anti-diabetic treatment [2–4]. Emerging studies have shown that stress hyperglycemia is essentially related to adverse outcomes in critically ill patients including nosocomial infection, acute kidney injury (AKI) onset, cardiovascular and cerebrovascular events, prolonged length of hospital stay, and mortality [5–8].

For critically ill patients with AKI, increased glucose levels at admission do not necessarily indicate stress-induced hyperglycemia [9], and the association between glycemic control and the adverse outcome has not been extensively investigated. Glycemic gap and stress hyperglycemia ratio (SHR) characterized by markers of acute-stress hyperglycemia could discriminate between acute and chronic hyperglycemia elevation in serum glucose values and were found to be independently correlated with poor outcomes in intensive care unit (ICU) patients [10]. Consequently, we hypothesized that in critically ill patients with AKI, the combination of acute and chronic glycemia may predict the adverse outcome with greater accuracy than single glycemia level. In the present study, we determined the relationship between acute and chronic glycemia and adverse events among critically ill patients with AKI.

## Methods

### Data source

The data were retrieved from Multiparameter Intelligent Monitoring in Intensive Care III version 1.4 (MIMIC-III v1.4), which is a large, publicly available critical care database [11]. It contains more than 40,000 critically patients who were admitted to Beth Israel Deaconess Medical Center. Access to database was approved by the institutional review boards of the Massachusetts Institute of Technology and the Beth Israel Deaconess Medical Center.  One author (LX) had access to this database (certification number 37,851,920) and was responsible for data extraction. All patients’ information was anonymized before extraction and data analysis, individual patient consent was waived. All methods complied with the relevant guidelines and recommendations.

### Population selection criteria

Adult patients (≥ 18 years) meeting criteria for AKI following the Kidney Disease Improving Global Outcomes (KDIGO) criteria at first ICU admission for more than 2 days were considered eligible for study inclusion. The KDIGO criteria were as follows [12]: serum creatinine increased by ≥ 0.3 mg/dl within 48 h or increased to ≥ 1.5 times from within the prior 7 days, or oliguria (urine volume < 0.5ml/kg/h) for 6 h or more. The first serum creatinine measured at ICU admission was used as the baseline serum creatinine when the preadmission serum creatinine was not available. Patients who met the following criteria were excluded: (1) no HbA1c or glucose measured at ICU admission; (2) discharged or died within 48 h; (3) missing > 5% potential risk variables for death.

## Data extraction

All patients’ data within first day after ICU admission were extracted from MIMIC-III using Structured query language (SQL) with PostgreSQL (version 9.6). Baseline characteristics were extracted, including age, gender, admission type, ethnicity, triglyceride (TG), total cholesterol (TC), hemoglobin A1c (HbA1c), serum creatinine, hemoglobulin, albumin, estimated glomerular filtration rate (eGFR), simplified Acute Physiology Score II (SAPSII), mean arterial pressure (MAP), use of vasopressors and insulin, AKI stage, renal replacement therapy (RRT) and ICU length of stay (LOS). The comorbidities were also collected, including chronic kidney disease (CKD), acute myocardial infarction (AMI), hypertension, diabetes mellitus (DM), chronic heart failure, chronic obstructive pulmonary disease (COPD), cancer, chronic liver disease and sepsis. The diagnosis of DM was made if this disease and/or current use of insulin or oral hypoglycemic agents, were recorded in the admission history. A diagnosis of unknown DM was made when patients had HbA1c ≥ 6.5% despite no previous history. Average chronic glucose levels were estimated by HbA1c, expressed as percentage value, according to the following validated equation: estimated chronic glucose levels (mg/dl) = 28.7 x HbA1c (%)-46.7 [13]. The stress hyperglycemia ratio (SHR) was defined as acute glucose (admission) divided by estimated chronic glucose levels [14]. The glycemic gap was defined as the absolute difference between acute and estimated chronic glucose [14]. Hyperglycemia was defined according to the American Diabetes Association (ADA) proposed threshold for in-hospital hyperglycemia as any blood glucose measurement > 140 mg/dl and hypoglycemia was any blood or capillary glucose < 70 mg/dl during the first day in the ICU [15, 16]. Acute glycemia was any blood or capillary glucose > 198 mg/dl during ICU stay [17]. The primary endpoint of our study was ICU all-cause mortality, the secondary outcome was need of RRT.

### Statistical analysis

Continuous variables were presented as mean ±standard deviation (SD) or median with interquartile range. Categorical variables were described as a number with percentage. Differences between groups were assessed using Pearson’s *X*^2^ test or the Fisher exact test for categorical variables and the independent *t*-test or Mann–Whitney U test for continuous variables, as appropriate. The association between glycemic parameters and mortality was assessed by logistic regression analysis. The clinical relevant variables at ICU admission and found to be associated with mortality at univariate analysis were enrolled in the multivariate model, including age, gender, eGFR, AKI stage, SAPSII and insulin treatment. Results are presented as odds ratios (OR) with 95% confidence interval (CI). The cutoff values of SHR and glycemic gap for mortality prediction were performed with the highest Youden index. Discrimination was calculated with areas under the curve (AUC), and AUC values were interpreted as follows: negligible (≤ 0.55), small (0.56–0.63), moderate (0.64–0.70) and strong (≥ 0.71) [18]. All statistical analyses were performed using SPSS 21.0 software (SPSS Inc., IBM, USA). A two-tailed value < 0.05 was considered statistically significant.

## Results

### Study population characteristics

A total of 2255 consecutive critically ill patients with AKI were included in the study. The baseline characteristics were summarized in Table 1. Briefly, the mean age of the entire population was 67 years, 63.4% were male and 62.2% of the subjects were white. The mean acute glucose level at ICU admission was 160.2 ± 93.6 mg/dl mg/d, and the estimated chronic glucose level was 140.4 ± 46.8 mg/d. The frequency of AKI stages 1,2 and 3 was 85.5%, 3.9%, and 10.6%, respectively.

**Table 1:** Baseline characteristics between survivors and non-survivors

	All patients (2255)	Survivors (*n* = 2000)	Non-survivors (*n* = 255)	*P* value
Age	67 ± 14	67 ± 14	69 ± 14	0.136
Male, n (%)	1429 (63.4)	1278 (63.9)	151 (59.2)	0.144
Ethnicity, n (%)				0.001
White	1403 (62.2)	1270 (63.5)	133 (52.2)	
Black	179 (7.9)	158 (7.9)	21 (8.2)	
Other	673 (29.8)	572 (28.6)	101 (39.6)	
Admission type, n (%)				0.001
Observation	299 (13.3)	269 (13.5)	30 (11.8)	
Elective	200 (8.9)	192 (9.6)	8 (3.1)	
Emergency	905 (40.1)	782 (39.1)	124 (48.6)	
Urgency	851 (37.7)	758 (37.9)	93 (36.5)	
MAP, mmHg	82.1 ± 18.8	82.1 ± 18.8	81.9 ± 19.2	0.849
Serum creatinine, mg/dl	1.7 ± 1.8	1.7 ± 1.9	1.8 ± 1.4	0.829
eGFR, mL/min/1.73m^2^	58.5 ± 30.6	59.5 ± 31.0	50.5 ± 27.1	< 0.001
HbA1c, %	6.5 ± 1.7	6.5 ± 1.7	6.4 ± 1.7	0.481
Acute glycemia, mg/dl	160.2 ± 93.6	158.4 ± 93.6	180.0 ± 100.8	0.001
Chronic glycemia, mg/dl	140.4 ± 46.8	140.4 ± 46.8	138.6 ± 48.6	0.482
SHR	1.2 ± 0.6	1.2 ± 0.6	1.3 ± 0.7	< 0.001
Glycemic gap, mg/dl	5.4 (-20.4, 44.4)	3.7 (-21.1, 40.6)	21.8 (-14.2, 75.9)	< 0.001
Hypoglycemia	32 (1.4)	26 (1.3)	6 (2.4)	0.181
Hyperglycemia	1016 (45.1)	864 (43.2)	152 (59.6)	< 0.001
Comorbidities, n (%)				
Diabetes	510 (22.6)	463 (23.2)	47 (18.4)	0.090
Hypertension	511 (22.7)	455 (22.8)	56 (22.0)	0.777
AMI	308 (13.7)	269 (13.5)	39 (15.3)	0.110
Heart failure	548 (24.3)	484 (24.2)	64 (25.1)	0.596
Chronic liver disease	62 (2.7)	49 (2.5)	13 (5.1)	0.015
Cancer	106 (4.7)	85 (4.3)	21 (8.2)	0.005
CKD	393 (17.4)	349 (17.5)	44 (17.3)	0.938
COPD	20 (0.9)	17 (0.9)	3 (1.2)	0.601
Scoring systems				
SAPSII	41.7 ± 13.6	40.7 ± 13.1	49.1 ± 15.1	< 0.001
AKI stage				0.048
Stage 1	1928 (85.5)	1697 (84.9)	231 (90.6)	
Stage 2	87 (3.9)	80 (4.0)	7 (2.7)	
Stage 3	240 (10.6)	223 (11.1)	17 (6.7)	
RRT use, n (%)	388 (17.2)	295 (14.8)	93 (36.5)	< 0.001
Vasopressin use, n (%)	968 (42.9)	809 (40.5)	159 (62.4)	< 0.001
Insulin use, n (%)	1737 (77.0)	1557 (77.9)	180 (70.6)	0.009
ICU LOS, day	7.8 ± 7.8	7.5 ± 7.5	10.4 ± 9.3	< 0.001

*HbA1c* Hemoglobin A1c, *eGFR* Estimated glomerular filtration rate, *COPD* Chronic obstructive pulmonary disease, *MAP* Mean artery pressure, *SHR* Stress hyperglycemia ratio, *SAPSII* Simplified acute physiology score II, *AKI* Acute kidney injury, *ICU* Intensive care unit, *LOS* Length of stay, *RRT* Renal replacement therapy

The overall mortality was 11.3%. Regardless of preexisting DM, the mortality rate of patients with and without DM was not significantly different (11.9% vs. 9.2%, *P* = 0.090). Non-survivors had significantly higher WBC, hemoglobin, anion gap, SAPS II score, higher rate of RRT support, and lower albumin and eGFR levels. Acute glycemia, glycemic gap and SHR were also significantly higher in non-survivors, but HbA1c and chronic glycemia were not. Moreover, non-survivors were more likely to have a history of chronic liver disease, cancer, ARDS, sepsis, and stroke, with a significant increase in the length of ICU stay.

## Association of glycemic parameters and clinical outcomes

**Fig. 1:**
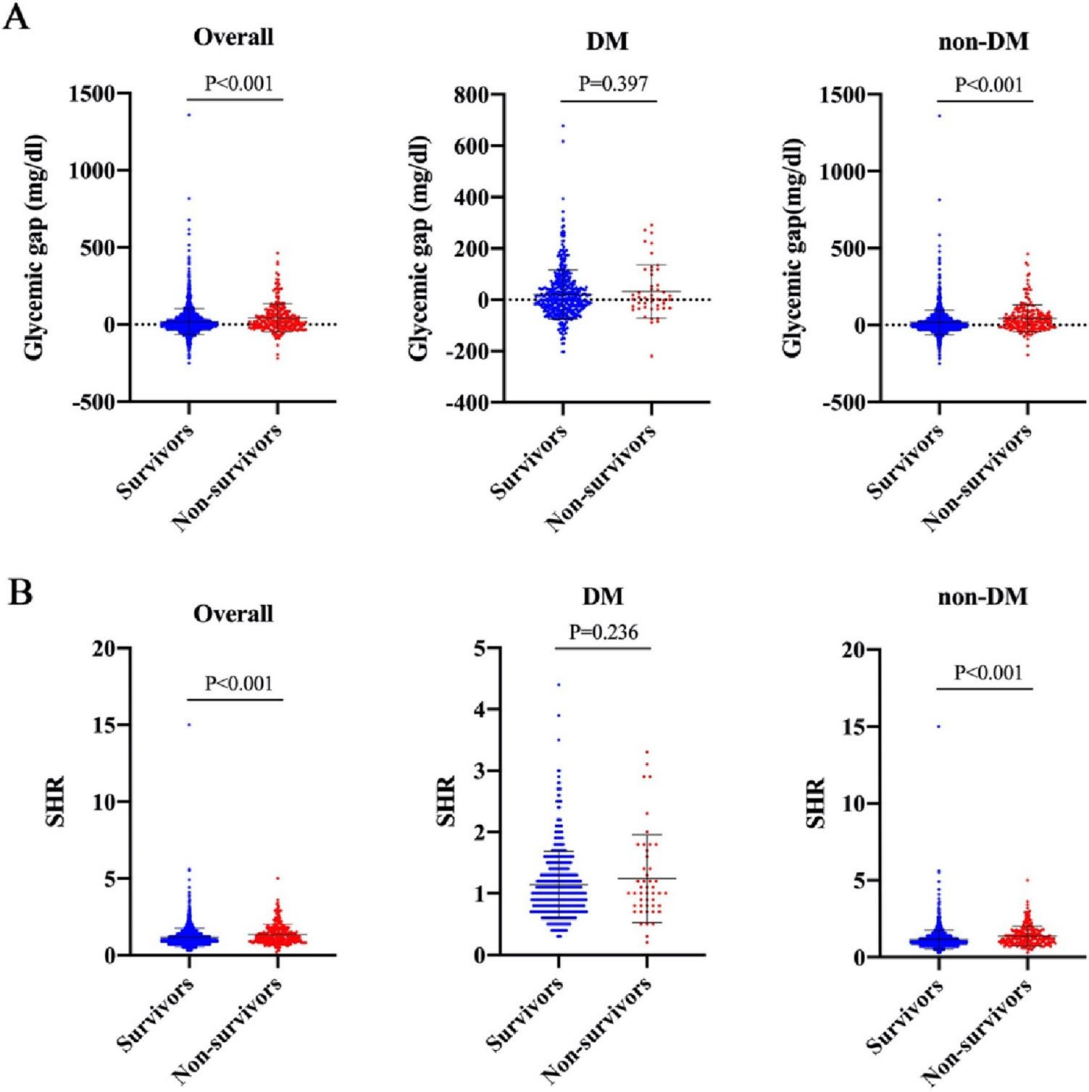
Glycemic gap (**A**) and stress hyperglycemia ratio (**B**) according to survival status in overall population, in patients with diabetes mellitus, and in patients without diabetes mellitus

The glycemic gap varied from − 250.4 to 1359.2 mg/dl, with a median value of 5.4 mg/dl in the overall population, with higher values in non-survivors. In subgroup analysis of preexisting DM, no significant difference was observed between survivors and non-survivors (*P* = 0.397) whereas glycemic gap was higher in non-survivors without DM (*P* < 0.001). Similarly, the SHR was found to be significantly higher in the non-survivors without DM (*P* < 0.001), but the difference is not demonstrated in patients with preexisting DM (*P* = 0.236) (Fig. 1).

The presence of hyperglycemia was associated with higher ICU mortality (15.0% vs. 8.3%, *P* < 0.001). Length of ICU stay was also prolonged in this population (8.3 ± 7.7 vs. 7.3 ± 7.8, *P* = 0.002). The presence of hypoglycemia within 24 h after ICU admission was associated with an increased need for RRT (37.5% vs. 16.9%, *P* = 0.002). At ROC analysis, the cutoff value of glycemic gap and SHR according to the highest Youden’s index was identified as 34.7 mg/dl and 1.2, respectively. Both glycemic gap ≥ 34.7 mg/dl and SHR ≥ 1.2 were associated with the greater need for higher mortality, RRT and longer ICU stay (Table 2).

**Table 2:** Effect of glycemic parameters on clinical outcomes

	Hypoglycemia			Hyperglycemia			SHR			Glycemic gap		
Outcomes	No *n*=2223	Yes *n*=32	*P*	No *n*=1239	Yes *n*=1016	*P*	<1.2 *n*=1350	≥1.2 *n*=905	*P*	<34.7 *n*=1586	≥34.7 *n*=669	*P*
RRT (n, %)	376 (16.9)	12 (37.5)	0.002	198 (16.0)	190 (18.7)	0.089	204 (15.1)	184 (20.3)	0.001	249 (15.7)	139 (20.8)	0.004
Mortality (n, %)	249 (11.2)	6 (18.8)	0.181	103 (8.3)	152 (15.0)	<0.001	115 (8.5)	140 (15.5)	<0.001	141 (8.9)	114 (17.0)	<0.001
LOS, ICU (days)	7.8±7.8	7.4±6.0	0.788	7.3±7.8	8.3±7.7	0.002	7.2±7.6	8.6±7.9	<0.001	7.4±7.7	8.6±8.0	0.001

*SHR* Stress hyperglycemia ratio, *RRT* Renal replacement therapy, *LOS* Length of stay, *ICU* Intensive care unit

The multivariate logistic regression analysis revealed that four glycemic parameters were associated with ICU mortality in the univariate analysis, and only hyperglycemia, glycemic gap and SHR remained associated with mortality after adjustments (OR 1.92, 95%CI 1.45–2.56, *P* < 0.001; OR 1.00, 95%CI 1.00-1.01, *P* = 0.019 and OR 1.30; 95%CI 1.05–1.61, *P* = 0.018, respectively) (Table 3). However, no glycemic parameter was associated with increased need for RRT. In addition, AKI stage, eGFR, SAPSII and insulin use were identified as independent risk factors in logistic regression analyses (Table S1).

**Table 3:** Prognostic effect of glycemic parameters on the risk of ICU outcomes

	Crude OR (95% CI)	*P* value	Adjusted OR (95% CI)	*P* value	AUC (95% CI)	*P* value
RRT						
Hypoglycemia	2.95 (1.43, 6.08)	0.002	1.95 (0.70, 3.45)	0.204	0.52 (0.49, 0.56)	0.142
Hyperglycemia	1.21 (0.97, 1.51)	0.089	0.76 (0.56, 1.02)	0.070	0.51 (0.48, 0.54)	0.530
Acute glycemia	1.02 (0.99, 1.04)	0.091	0.93 (0.82, 1.25)	0.287	0.52 (0.49, 0.55)	0.223
Chronic glycemia	0.93 (0.88, 0.97)	0.002	1.10 (0.81, 1.49)	0.550	0.44 (0.40, 0.47)	< 0.001
Glycemic gap	1.39 (1.17, 1.66)	< 0.001	1.00 (0.99, 1.00)	0.160	0.56 (0.53, 0.59)	< 0.001
SHR	1.01 (1.00, 1.01)	0.001	0.92 (0.75, 1.12)	0.405	0.55 (0.52, 0.59)	0.001
ICU mortality						
Hypoglycemia	1.83 (0.75, 4.49)	0.187	1.68 (0.65, 4.33)	0.283	0.51 (0.47, 0.54)	0.784
Hyperglycemia	1.94 (1.49, 2.53)	< 0.001	1.92 (1.45, 2.56)	< 0.001	0.58 (0.55, 0.62)	< 0.001
Acute glycemia	1.04 (1.02, 1.06)	0.001	1.18 (0.89, 1.56)	0.244	0.59 (0.55, 0.62)	< 0.001
Chronic glycemia	0.98 (0.93, 1.03)	0.482	1.00 (0.94, 1.06)	0.915	0.48 (0.45, 0.52)	0.378
Glycemic gap	1.50 (1.23, 1.82)	< 0.001	1.00 (1.00, 1.01)	0.019	0.60 (0.56, 0.63)	< 0.001
SHR	1.01 (1.00, 1.02)	< 0.001	1.30 (1.05, 1.61)	0.018	0.60 (0.56, 0.63)	< 0.001

OR was adjusted for age, gender, ethnicity, ICU admission, AKI stage, SAPSII, insulin administration, eGFR values and ICU mortality in the multivariate model

*ICU* Intensive care unit, *SHR* Stress hyperglycemia ratio, *eGFR* estimated glomerular filtration rate, *RRT* Renal replacement therapy, *SAPS II* Simplified acute physiology score II, *OR* Odds ratio, *CI* Confidence interval

## Subgroup analysis based on patients with and without diabetes

In non-diabetic critically ill patients with AKI, glycemic gap and SHR over identified cutoff values were significantly associated with ICU mortality, even after adjusting for clinical confounding factors (OR 2.25, 95%CI 1.64–3.08, *P* < 0.001 and OR 1.99; 95%CI 1.46–2.72, *P* < 0.001, respectively). However, the glycemic gap and SHR could not predict ICU mortality in patients with diabetes (Table 4).

**Table 4:** Subgroup analysis based on patients with and without diabetes

Subgroup	Glycemic gap ≥ 34.7		SHR ≥ 1.2	
	OR (95%CI)	*P*	OR (95%CI)	*P*
RTT				
Non-diabetic	1.02 (0.73, 1.45)	0.895	1.17 (0.84, 1.62)	0.360
Diabetic	0.76 (0.39, 1.49)	0.418	0.75 (0.39, 1.44)	0.750
ICU mortality				
Non-diabetic	2.25 (1.64, 3.08)	<0.001	1.99 (1.46, 2.72)	<0.001
Diabetic	0.93 (0.48, 1.80)	0.821	0.92 (0.48, 1.75)	0.797

OR was adjusted for age, gender, ethnicity, AKI stage, SAPSII, insulin administration and eGFR values in the multivariate model

*ICU* Intensive care unit, *SHR* Stress hyperglycemia ratio, *eGFR* estimated glomerular filtration rate, *RRT* Renal replacement therapy, *OR* Odds ratio, *CI* Confidence interval

## The predictive value of the combination of glycemic parameters and SAPSII with ICU mortality

**Fig. 2:**
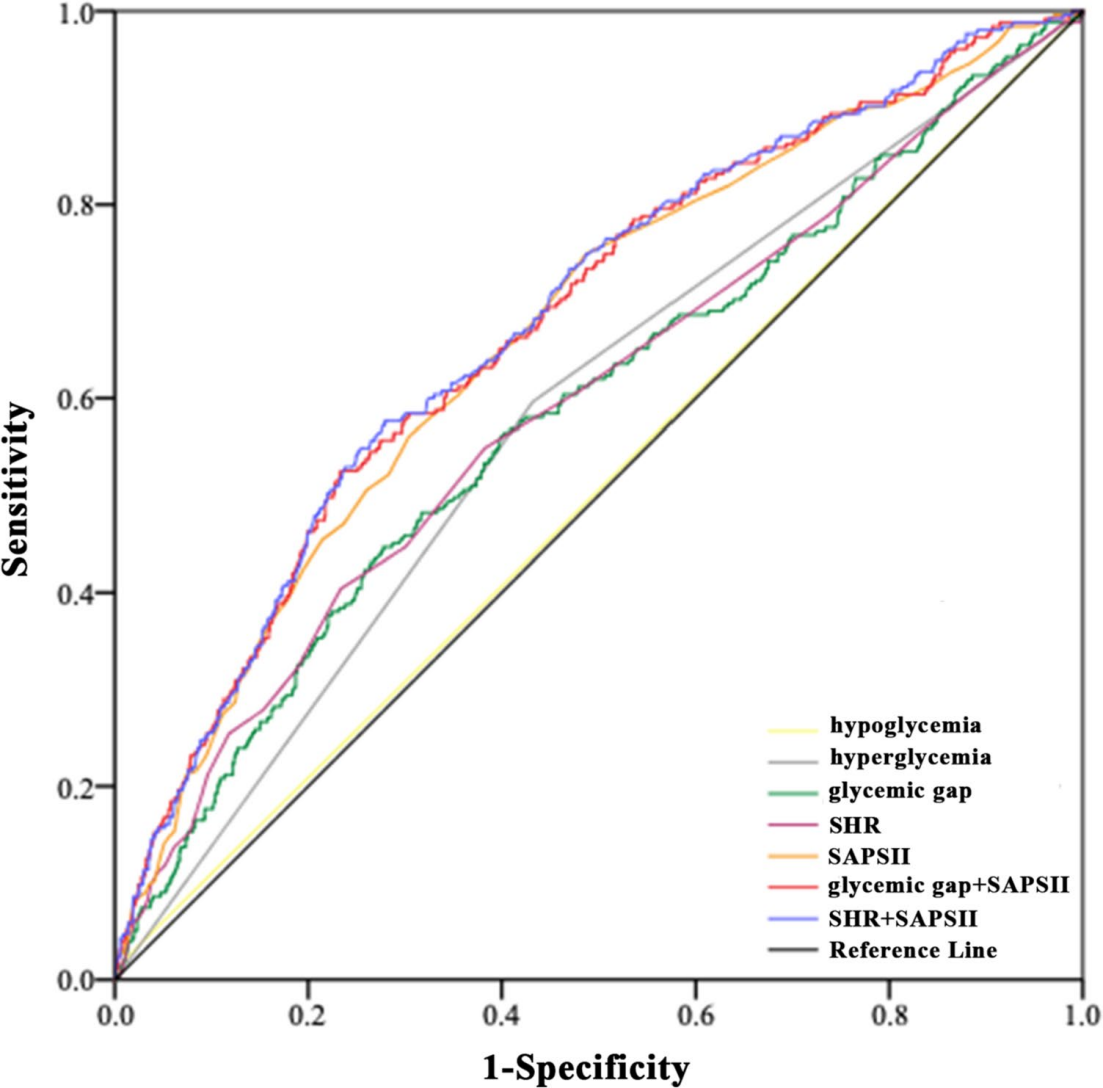
Predictive value of glycemic parameters, SAPSII and their combinations for ICU mortality in critically ill patients with AKI

The ROC curves indicated that the discriminatory power of glycemic gap and SHR for ICU mortality were small (Table 3). The AUC for SAPSII, glycemic gap plus SAPSII and SHR plus SAPSII were 0.668, 0.677 and 0.681, respectively (Fig. 2).

## Discussion

The main finding of our study was that the predictive value of glycemia at admission for ICU mortality in critically ill patients could be partially improved when the average chronic glycemia, as calculated by HbA1c, was also considered. Additionally, we found that both glycemic gap and SHR, instead of admission glycemia alone, were related to poor ICU outcomes, even after adjustment for important confounders.

Acute hyperglycemia is a strong predictor of adverse outcomes in critically ill patients with and without DM [19, 20]. Previous studies revealed that the use of continuous insulin infusion to keep the blood glucose level between 80 and 100 mg/dl reduced ICU mortality in critically ill patients [21–23]. However, the results based on the NICE-SUGAR study recommend that targeted blood glucose level control for most ICU patients at 140-180 mg/dl, resulting in reduced hypoglycemia complication but without affecting ICU mortality [24, 25]. In our study, the presence of hyperglycemia was independently associated with ICU mortality. However, a knowledge gap exists regarding the association between high glucose levels and worse outcomes. The possible explanations could be that the impaired immune system function and increased proteolysis induced by hyperglycemia may result in increased occurrence of infectious complications and ICU-acquired muscular weakness respectively [26–29]. Furthermore, in the present study, the mortality rates of patients with preexisting DM were similar to those of patients without, which is in line with the concept of the “diabetic paradox” in the ICU that diabetes is not independently correlated with increased mortality risk in the heterogeneous population of critically ill patients [30].

Glycated hemoglobin and blood glucose levels are generally recognized as biomarkers for glycemic targets in ICU care worldwide. Despite the lack of consensus with regard to appropriate definitions and tools for its assessment, glycemic variability seems to have a more detrimental influence than sustained hyperglycemia in the pathogenesis of diabetic CV complications [31, 32]. Thus, growing evidence demonstrated that glycemic variability indexes improved diagnostic and prognostic performance compared with standard glycemic parameters [33]. The glycemic gap and SHR are indexes of stress-induced hyperglycemia as they can distinguish glycemic variation from critical illness or metabolic status. The prognostic power of stress-induced hyperglycemic markers was more prominent than the absolute blood glucose level in acute illness patients such as acute myocardial infarction, sepsis and stroke [34–36]. Furthermore, a strong relationship between stress hyperglycemia and renal injury has been identified for decades. A rapid glycemic rise can cause osmotic diuresis and therefore lead to volume depletion and dehydration [37]. Moreover, acute hyperglycemia directly acerbates inflammation and oxidative stress, which may further reduce renal reperfusion [38]. All of these pathophysiologic changes could contribute to the development of renal complications and subsequently result in a poor prognosis.

The aim of the present study was to investigate the role of the glycemic gap and SHR in critically ill patients with AKI. In our selected population, hyperglycemia was independently associated with ICU mortality. It was proposed that the deleterious effects of hyperglycemia on immune systems and increased proteolysis resulted in lean tissue breakdown [39], which might contribute to the increased risk of infection and ICU-acquired muscular weakness, respectively [40, 41]. Compared with HbA1c variation and fasting blood glucose, the glycemic gap is a simple and reliable parameter for glucose variability, absolute acute glycemic rise (glycemic gap) was found to predict adverse outcomes [42, 43]. Consistently, we observed a significant increase in ICU mortality in patients with glycemic gap and SHR above the identified cutoff values, and the association remained significant even after adjustment for important clinical confounders. The explanation might be that neither acute nor chronic glycemic levels could necessarily reflect a genuine glycemic alternation in response to acuter illness, especially in diabetic patients with progressively elevated blood glucose levels, thus the performance of acute and chronic glycemic levels for ICU mortality prediction could be attenuated in DM. The previous experimental and clinical evidence on AKI demonstrated that acute glycemic changes have a detrimental effect on renal function compared with absolute glycemic levels [44]. Accordingly, it has been demonstrated that intensive lowering of glycemia may have a detrimental effect in patients with high glycemic values upon admission but without stress hyperglycemia [45]. Conversely, we were not able to identify which combination of acute and chronic glycemia was more closely correlated with AKI risk. The glycemic gap and SHR were not associated with an increased need for RRT. A possible explanation could be that our sample represents a mild to moderate renal impairment population, which reduced the risk of the need for RRT.

Several studies investigated whether a higher glycemic gap and SHR were also risk factors for mortality in a general population of ICU patients but yield conflicting results. In the present study, we observed that the relationship between a higher glycemic gap and SHR with ICU mortality was only apparent in non-diabetic critically ill patients with AKI, which stand in contrast to the finding of previous studies on the glycemic gap and SHR in very specific population critically ill patients with diabetes [14, 46]. One possible explanation could be an intensive glucose-lowering treatment can markedly increase the risk of hypoglycemia, the strict glycemic control strategies are proven to have neutral or even deleterious effects on cardiovascular outcomes in diabetic critically ill patients [37, 47, 48]. Therefore, a higher glycemic gap and SHR would be necessary for critically ill patients with DM. Interestingly, a large retrospective study demonstrated that in critically ill patients with and without DM, the glycemic gap and SHR were independently associated with adverse outcomes, but not with mortality [11], indicating a complex relationship between glucose variability and clinical risk in this patient population. The reasons for the discrepancy between the glycemic gap and SHR in patients with and without diabetes are not clear. Thus, whether the glycemic gap and SHR are of good predictive significance in patients requiring intensive care could be further investigated with longer follow-up.

Some limitations should be acknowledged. First, our study was conceivably underpowered to detect an effect of stage 3 AKI on ICU mortality and need for RRT, as only 240 patients (10.6%) with stage 3 AKI were enrolled. Second, several glycemic variabilities were not investigated in our research, such as maximal glycemic difference or glycemic lability index, we cannot compare which one is better parameter related to ICU mortality, however, our interesting glycemic gap and SHR were practical and more easily applicable in the common clinical practice. Third, information on enteral nutrition and on dose of insulin therapy were not recorded, thus the impact of carbohydrate intake and insulin administration on outcomes should be taken into account as possible biases. Forth, the glycemic gap and SHR were estimated from HbA1c, we cannot exclude the possibility that these indexed do not completely reflect acute glycemic change during ICU stay. Finally, although the main clinical relevant variables were adjusted to the multivariate model and subgroup analysis was performed, some potential residual confounding factors might influence the results were not analyzed. The prospect of the glycemic gap and SHR would be promising for its applicability and effectiveness, but far from claiming superiority due to the predictive accuracy of these novel markers are still moderate and their performance should be further investigated by external validation.

In conclusion, we demonstrated that in non-diabetic critically ill patients with AKI, the glycemic gap and SHR were more closely associated with ICU mortality than admission glycemia alone. The assessment of the glycemic gap and SHR might support early identification of critically ill patients with AKI at high risk of mortality who will benefit from intensive lowering treatment.

## Supplementary Information

**Additional file 1:** **Table S1.** Unadjusted and adjusted ORs of risk factors for ICU outcomes.

## Data Availability

The clinical data used to support the findings of this study was supplied by Monitoring in Intensive Care Database III version 1.4 (MIMIC-III v.1.4). Although the database is publicly and freely available, researchers must complete the National Institutes of Health’s web-based course known as Protecting Human Research Participants to apply for permission to access the database. The datasets used and analyzed during the current study are available from the corresponding author on reasonable request.
