# Supplementary material for: Predictive value of glycemic gap and stress glycemia ratio among critically ill patients with acute kidney injury: a retrospective analysis of the MIMIC-III database
Source: BMC Nephrol. 2023 Aug 1;24:227. doi: 10.1186/s12882-023-03278-z (PMC10394760; doi:10.1186/s12882-023-03278-z)
Supplement: Supplementary file 1 — Additional file 1: Table S1. Unadjusted and adjusted ORs of risk factors for ICU outcomes. [file 12882_2023_3278_MOESM1_ESM.docx]

Table S1. Unadjusted and adjusted ORs of risk factors for ICU outcomes

|  | Crude OR (95% CI) | *P* value | Adjusted OR (95% CI) | *P* value |
| --- | --- | --- | --- | --- |
| RRT |  |  |  |  |
| Age | 0.98 (0.97-0.99) | <0.001 | 0.96 (0.95-0.97) | <0.001 |
| Gender (male) | 1.00 (0.80-1.25) | 0.989 |  |  |
| Ethnicity |  |  |  |  |
| White | 1 |  |  |  |
| Black | 1.44 (0.97-2.13) | 0.071 |  |  |
| Other | 1.54 (1.22-1.95) | <0.001 | 1.44 (1.04-1.99) | 0.028 |
| AKI stage |  |  |  |  |
| 1 | 1 |  |  |  |
| 2 | 0.74 (0.34-1.62) | 0.452 |  |  |
| 3 | 4.49 (3.85-5.24) | <0.001 | 5.77 (3.68-9.02) | <0.001 |
| eGFR | 0.94 (0.93-0.95） | <0.001 | 0.97 (0.96-0.97) | <0.001 |
| SAPSII | 1.06 (1.05-1.07) | <0.001 | 1.05 (1.04-1.06) | <0.001 |
| Insulin use | 0.86 (0.67-1.11) | 0.240 | 1.01 (0.71-1.46) | 0.944 |
| ICU mortality |  |  |  |  |
| Age | 1.01 (0.99-1.02) | 0.137 |  |  |
| Gender (male) | 1.22 (0.93-1.59) | 0.134 |  |  |
| Ethnicity |  |  |  |  |
| White | 1 |  |  |  |
| Black | 1.27 (0.78-2.07) | 0.339 |  |  |
| Other | 1.69 (1.28-2.22) | <0.001 | 1.43 (0.82-2.51) | 0.207 |
| AKI stage |  |  |  |  |
| 1 | 1 |  |  |  |
| 2 | 0.64 (0.29-1.41) | 0.270 |  |  |
| 3 | 1.79 (1.07-2.98) | 0.026 | 3.15 (1.71-5.89) | <0.001 |
| eGFR | 0.99 (0.98-1.00) | <0.001 | 0.99 (0.98-1.00) | 0.011 |
| SAPSII | 1.04 (1.03-1.05) | <0.001 | 1.03 (1.02-1.05) | <0.001 |
| Insulin use | 0.68 (0.51-0.91) | 0.010 | 0.66 (0.47-0.92) | 0.015 |

﻿﻿ICU intensive care unit; eGFR estimated glomerular filtration rate; RRT renal replacement therapy; SAPS II simplified acute physiology score II; OR odds ratio; CI confidence interval
